# Technological Response of Wild Macaques (*Macaca fascicularis*) to Anthropogenic Change

**DOI:** 10.1007/s10764-017-9985-6

**Published:** 2017-08-29

**Authors:** Lydia V. Luncz, Magdalena S. Svensson, Michael Haslam, Suchinda Malaivijitnond, Tomos Proffitt, Michael Gumert

**Affiliations:** 1 School of Anthropology and Museum Ethnography, University of Oxford, Oxford, OX2 6PE UK; 2 School of Archaeology, University of Oxford, Oxford, OX1 2PG UK; 3 Department of Social Science, Oxford Brookes University, Oxford, OX3 0BP UK; 4 Department of Biology, Faculty of Science, Chulalongkorn University, Bangkok, 10330 Thailand; 5 National Primate Research Center of Thailand, Chulalongkorn University, Saraburi, Thailand; 6 School of Humanities and Social Sciences, Nanyang Technological University, Singapore, 637332 Singapore

**Keywords:** Anthropogenic influence, Behavioral flexibility, *Macaca fascicularis*, Nut cracking, Tool use

## Abstract

Anthropogenic disturbances have a detrimental impact on the natural world; the vast expansion of palm oil monocultures is one of the most significant agricultural influences. Primates worldwide consequently have been affected by the loss of their natural ecosystems. Long-tailed macaques (*Macaca fascilularis*) in Southern Thailand have, however, learned to exploit oil palm nuts using stone tools. Using camera traps, we captured the stone tool behavior of one macaque group in Ao Phang-Nga National Park. Line transects placed throughout an abandoned oil palm plantation confirmed a high abundance of nut cracking sites. Long-tailed macaques previously have been observed using stone tools to harvest shellfish along the coasts of Thailand and Myanmar. The novel nut processing behavior indicates the successful transfer of existing lithic technology to a new food source. Such behavioral plasticity has been suggested to underlie cultural behavior in animals, suggesting that long-tailed macaques have potential to exhibit cultural tendencies. The use of tools to process oil palm nuts across multiple primate species allows direct comparisons between stone tool using nonhuman primates living in anthropogenic environments.

**Electronic supplementary material:**

The online version of this article (10.1007/s10764-017-9985-6) contains supplementary material, which is available to authorized users.

## Introduction

The rapid expansion of humans across the globe has affected almost every part of the natural environment. Large-scale processes, such as increases in pollution and rates of climate change, may be combined with more local effects, including habitat destruction, and the introduction of monocultures and exotic species (Sih 2013). These anthropogenic effects can have negative outcomes for animals, especially if the effects are so rapid or extensive that biological adaptations such as phenotypic changes or niche modifications are insufficient to create adaptations due to natural selection (Jezkova and Wiens 2016). One potential buffer is behavioral plasticity, which permits individuals to adjust rapidly to local changes ahead of any phenotypic adjustments (Slabbekoorn and Ripmeester 2008). Anthropogenic changes in the natural habitat of animals can therefore also be used to understand behavioral flexibility and cognition. Behavioral responses to environmental changes can also provide guidelines for conservation efforts (Hockings *et al.* 2015).

A common human response to environmental threats has been to search for behavioral, and more specifically technological, solutions (Hoffert *et al.* 2002). We have little evidence that other tool-using species have followed a similar path of increased tool use in the face of anthropogenic change. More commonly, on the contrary, human activities negatively affect tool-using taxa. For example, logging is one potential cause of the absence of stick tool use among chimpanzees (*Pan troglodytes schweinfurthii*) of the East African Sonso community (Gruber 2013).

Tool use is rare in nonhuman primate species, and stone tools are used only by some groups of Western chimpanzee (*Pan troglodytes verus*: Boesch and Boesch 1983; Matsuzawa *et al.* 2011), bearded capuchin monkeys (*Sapajus libidinosus*: Fragaszy *et al.* 2004; Proffitt *et al.* 2016), and long-tailed macaques (*Macaca fascilularis*: Gumert and Malaivijitnond 2012; Malaivijitnond *et al.* 2007). Stone tools are invaluable when studying responses to changing conditions over time, as they leave long-lasting evidence in the home range of the respective species, preserving past behaviors in the habitat (Luncz *et al.* 2015). Using modern archaeological techniques, it is becoming possible to interrogate the past tool-using habits of these primate species (Carvalho and McGrew 2012; Haslam et al. 2009, 2016a, 2016b).

Long-tailed macaques (*Macaca fascicularis*) are omnivorous primates with high dietary plasticity that exploit a wide variety of shellfish, nuts, and other encased food in coastal areas of Thailand and Myanmar (Gumert *et al.* 2009; Gumert and Malaivijitnond 2012). The variety of prey targeted by long-tailed macaques has been observed to exceed that seen in other tool-using primates (Gumert *et al.* 2009; Tan *et al.* 2015). The arrival of anthropogenic factors such as farming and domesticated dogs has, however, disrupted access to suitable food items, resulting in threats to macaque tool use traditions (Gumert *et al.* 2013).

Oil palm nuts (*Elaeis guineensis*) are considered a valuable high-calorie food source rich in proteins, fat, and vitamins (Sundram *et al.* 2003). The global expansion of oil palm monocultures has led to widespread clearing of natural forests worldwide, with an accompanying loss of species richness (Fitzherbert *et al.* 2008). Here we report technological oil palm exploitation by island-dwelling wild macaques in Ao Phang-Nga National Park, Thailand. Macaques in this park live in a hybrid zone of common (*Macaca fascicularis*) and Burmese (*M. F. aurea*) long-tailed macaques (Bunlungsup *et al.* 2016; Fooden 2006). This group has learned to crack open oil palm nuts using stone tools, following human introduction of oil palm into their habitat in the early 2000s. The recent inception of this behavior offers a valuable primatological and archaeological resource to document directly the development of a primate tool use behavior in a new ecological setting.

## Methods

**Fig. 1:**
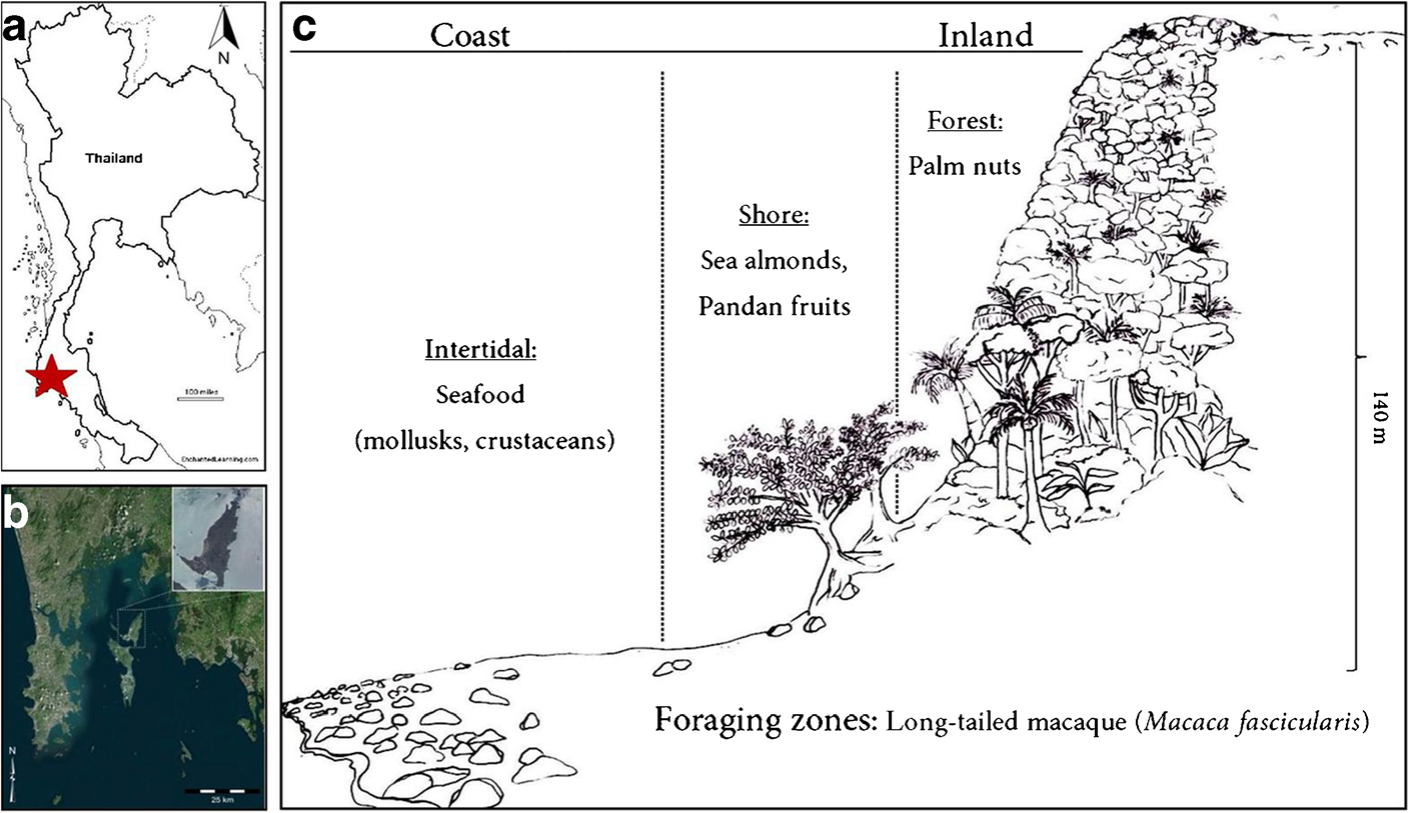
Study site on Yao Noi Island. **a** Field site. Location of Ao Phang-Nga National Park in Thailand. **b** Location of Yao Noi Island within the park. **c** Schematic illustration of topographic tool-assisted foraging zones of long-tailed macaques in Thailand. (Illustrator: Kathleen Reinhardt).

We conducted fieldwork in February and October 2016 on Yao Noi Island in Ao Phang-Nga National Park, Thailand (Fig. 1b). Our field site, Lobi Bay, is located on the northern shore of the island (8°10.838′N, 98°37.746′E). Yao Noi Island is a hilly tropical forest island with an area of ca. 45 km^2^. The research site consists of an intertidal zone, a sandy beach ca. 5 m wide, and steep coastal forest (Fig. 1b). Within the forest there is an abandoned and overgrown oil palm plantation, the trees of which still regularly produce fruits. Both long-tailed and pig-tailed macaques (*Macaca leonina*) range within the field site.

**Fig. 2:**
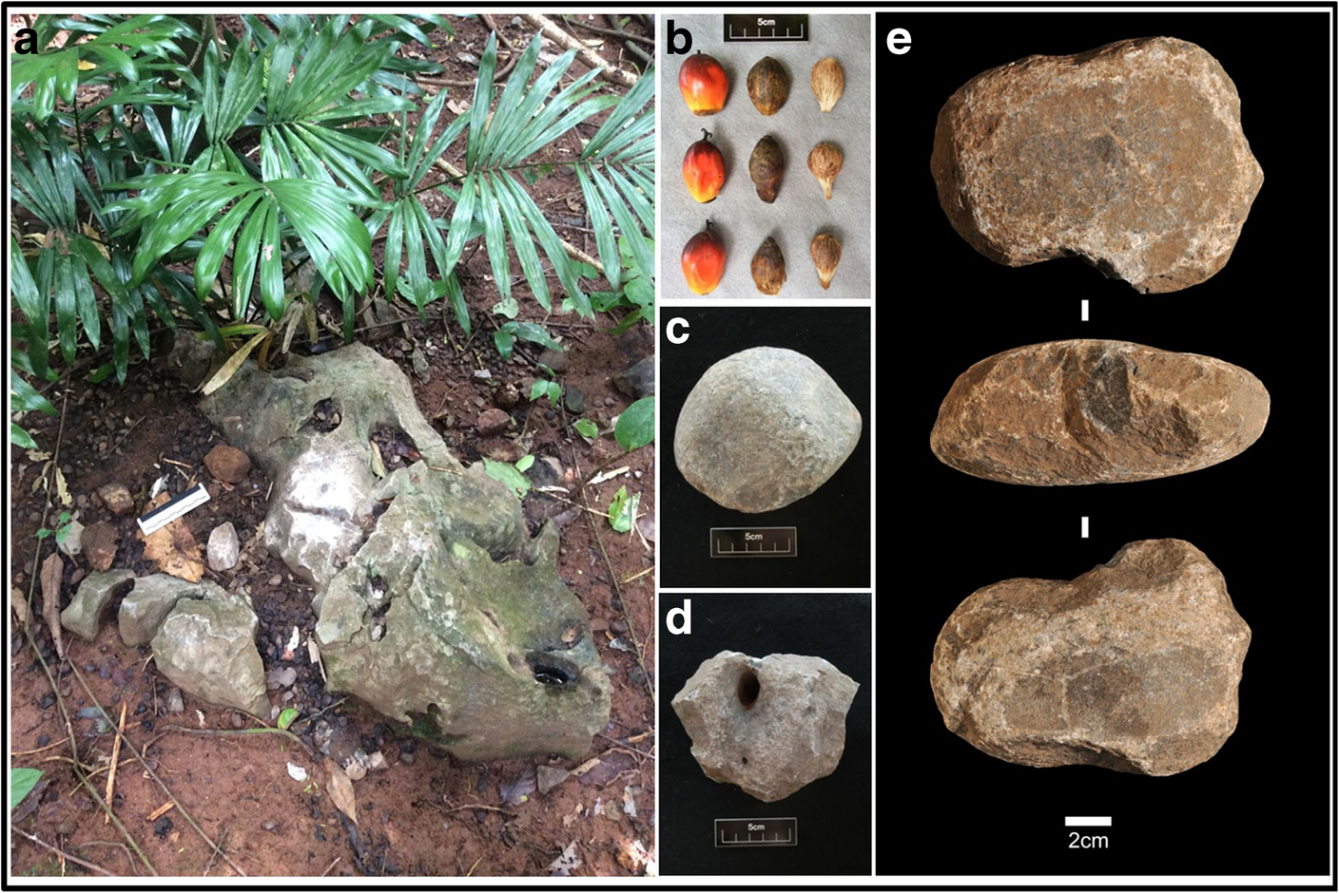
Macaque oil palm nut cracking site. **a** Used anvil, with stone hammerstone assemblages. **b** Three different ripeness stages of oil palm nuts consumed by macaques. **c, d, e** Used hammerstones.

The long-tailed macaques at Lobi Bay are not habituated to the presence of humans. Initial observations of these macaques confirmed frequent shellfish cracking using stone tools along the intertidal shoreline. In addition, they use stones to crack open sea almonds (*Terminalia catappa*) along the shore, above the intertidal zone, as seen at other macaque tool use sites (Falotico *et al.* 2017). Following initial findings of potential macaque oil palm nut-cracking sites with broken nutshells and stone hammers at anvils (Fig. 2), we set up 12 Bushnell HD trophy camera traps at 10 previously used anvils within the abandoned oil palm plantation, distributed from the shoreline up to ca. 120 m above sea level. At each camera site/anvil, we provided oil palm nuts from nearby active plantations, as the oil palm trees at the field site were not producing nuts at the time of the study. We visited the sites every second day and provided new nuts whenever they were depleted.

To assess the availability and densities of raw materials, tool numbers, and oil palm trees we placed line transects within the abandoned oil palm plantation that was included in, but did not equal, the home range of the macaque group under study. The plantation was located within steep terrain, pervaded with cliffs and canyons. To survey the maximum area in the abandoned palm oil plantation, we arranged line transects in a zig-zag pattern, with insurmountable obstacles dictating the number of changes of direction in our line transects. Whenever a dead end was encountered we altered the direction of the transect by 45 degrees and continued sampling. This method resulted in a total of 13 line transects, surveying a total length of 1600 m.

We defined hammerstones as portable rocks with clear use-wear in the form of central depressions, and use damage along their edges, found within a maximum distance of 50 cm of an anvil (Fig. 2a, c–e). We defined anvils as fixed limestone outcrops with traces of use on their horizontal planes, and broken nut shells located within 50 cm circumference (Fig. 2a). We recorded hammerstone weight, dimensions, and material within 1 m either side of the transects, as well as anvils and their distance to the nearest nut tree. To compare weights between tools we log-transformed the weights due to a strong left skewed distribution of the samples. We also estimated the density of oil palm trees by counting their abundance within 5 m either side of the transect. To calculate densities, we used the formula *D* = *n*/2*Lw*, where *n* = number of objects, *L* = total length of transect, and *w* = strip width.

## Ethical Note

Research was conducted using noninvasive observational data collection on natural nut cracking behavior in wild macaques. This research has been approved by the Institutional Animal Care and Use Committee, NTU, Singapore (ARF SBS/NIE-A0210). The Department of National Parks, Wildlife and Plant Conservation issued the permission to conduct research in the National Park (Permit Reference Number: DNP0907.4/22663, issued on October 31, 2016; the valid duration is October 1, 2016–September 30, 2018). In addition, the research adhered to the legal requirements of Thailand.

## Results

All camera traps combined recorded a total of 529 min (8 h and 49 min) of macaque oil palm nut cracking behavior over 21 days. Once the macaques realized that nuts were available at the anvils they visited almost every day. Each camera trap frequently recorded tool use of long-tailed macaques. The footage demonstrated that the group in the monitored home range consisted of both male and female macaques of all age classes. As the group is not habituated to human presence we were not able to clearly identify individuals and precise evaluation of group size therefore was not possible. However, the average macaque group usually has ca. 20–30 individuals (Fooden 1995), and our footage suggests that the observed group ranges within these numbers too. Adult and juvenile macaques of both sexes consumed oil palm nuts, as we are not yet able to identify individuals we conservatively will classify this behavior as habitually (sensu Whiten *et al.* 2001). The macaques first ate the oily mesocarp and then used a stone to crack the inner kernel-bearing shell. To do so, they selected a stone hammer near the anvil, placed one nut at a time on top of the anvil and struck the nut until it broke (Electronic Supplementary Material (ESM), Video S1). They then used their fingers and occasionally their teeth to extract the kernel from the broken shell. The obtained video footage suggests that tool use by adult group members attracts the interest of infants and juveniles, as they often were filmed in proximity to a tool user, closely watching the feeding behavior.

Whenever the macaques triggered the camera traps, tool use was recorded. This suggests that macaques visited the sites only for nut cracking. We never recorded an alternative extraction method of oil palm that did not use tools.

Available stones, used hammerstones, and used anvils were abundant along the line transects (Table I, Fig. 2). Following our definitions, objects identified as hammerstones and anvils showed clear percussive marks. The primary raw material for hammerstones was limestone (*N* = 252; 74%), followed by laterite (*N* = 73; 22%) and granite (*N* = 13; 4%), mirroring the availability of these stone types on the landscape (Table I). Hammerstones and unused stones did not significantly differ in weight (*t* = −0.56, df = 171.43, *P* = 0.57, log-transformed due to a strong left skewed distribution of the samples). However, every granite stone we encountered had been used by the macaques. Overall, the macaques used a high percentage (*N* = 338; 75%) of the available stones as hammers. We located a mean of 1.61 hammerstones for each anvil (ESM S2).

**Table I:** Encounter rates of wild macaque lithic technology on line transects, Lobi Bay, Yao Noi Island, Thailand

	Stones (total)	Hammer-stones	Stones (unused)	Boulders available (total)	Anvils (used)	Unused boulders	Oil trees
Total no.	448	338	110	251	210	41	66
Limestone	350	252	98	238	197	41	
Laterite	85	73	12	12	12	0	
Granite	13	13	0	0	0	0	
Wood	—	—	—	1	1	—	
Density (m^2^)	0.140	0.105	0.034	0.078	0.065	0.013	0.004
Measurements	Weight (g)	Weight (g)	Weight (g)	Horizontal area (cm^2^)	Horizontal area (cm^2^)	Horizontal area (cm^2^)	Distance anvil to tree (m)
Median	206	217.5	195	357	390	120	5
Range	56–5000	59–5000	56–3500	21–17,600	30–17,600	21–1488	0.1–20
1st–3rd quartile	136–415.5	136–411	133–422	162.5–828.5	208–964	56–304	0.1–7.75

Stone anvils were almost exclusively unmovable limestone outcrops (*N* = 197; 94%), with the remainder formed of laterite (*N* = 12; 6%). The median distance from an anvil to the nearest tree was 5 m (ESM S2). We found one wooden anvil, with a distinct depression the size of an oil palm nut, during the transect surveys. Off-transect surveys around the identified nut-cracking sites located another two wooden anvils. Palm oil trees occurred at a density of one every 250 m^2^ along the transects.

## Discussion

Our study showed that wild macaques on Yao Noi Island in the Ao Phang-Nga National Park frequently use stone tools to crack open oil palm nuts in an abandoned plantation. The tools selected for nut-cracking did not differ significantly from unused stone material found in the territory. This suggests that the macaques do not have a strong preference for a specific nut-cracking tool size. The high abundance of used tools and anvils indicates that the macaques are consuming this food source at a significant level, even though the arrival of oil palm nuts in Yao Noi Island is a recent event.

Dietary plasticity has been proven to be beneficial for long-tailed macaques, making them one of the most successful primate species, living in both undisturbed and urban areas where they exploit anthropogenic food sources (Gumert 2011). As part of this plasticity, macaques have been known for more than 100 years to use lithic technology (Carpenter 1887). At Lobi Bay, oil palm nuts were reportedly introduced by humans around 13 years ago (ca. 2004). Compared to their known exploitation of marine prey, this is a relatively short time span for the macaques to learn to exploit this novel food source. We hypothesize that long-tailed macaques have transferred an existing technological solution of harvesting encased shellfish within a coastal environment to exploit the nutritious nut of palm oil trees within an inland setting. Although currently benefiting from this new food source, oil palm nut cracking could potentially affect their natural foraging patterns on the coast. Future investigations will need to identify how this new tool use behavior influences the wider macaque range of tool-assisted foraging patterns, including its potential to diminish their natural sea food consumption and associated tool diversity.

The abundant and long-term evidence of macaque stone tool use in coastal settings (Carpenter 1887; Gumert and Malaivijitnond 2012; Haslam *et al.* 2016b; Malaivijitnond *et al.* 2007) suggests that stone tool use originated in the intertidal zone before expanding into the adjacent forest (Fig. 1c). Although this behavior is therefore likely to be relatively new in the tool repertoire of wild macaques, it offers the potential to study fine grained diachronic patterns of primate tool use, such as modification of the technology (choice of hammerstone size, morphology, raw material) and spatial and chronological development of the behavior. From a primate archaeological perspective this discovery is also significant, as this macaque behavior leaves a substantial and discrete archaeological record, one that is not affected by a daily intrusion from tidal forces. It therefore provides a new archaeological target for understating how primate tool use develops in relation to a sudden change of food source (Potts 2013). Further archaeological investigations are needed to assess whether the behavior was initiated close to the shore (where percussive activity is prevalent) and then spread inland, or whether it appeared across all oil palm producing regions essentially simultaneously. The effect and responses of wildlife to anthropogenic changes are sometimes difficult to observe directly when they are happening. Therefore, archaeological methods may help to reveal the potential loss or gain of a behavior by investigating past tool use. Future work must therefore focus on the targeted excavation of macaque oil palm nut cracking locations across the landscape.

The rapid uptake of oil palm nut cracking by the Yao Noi Island macaques shows their ability to take advantage of anthropogenic changes, and the recent establishment of this behavior indicates the potential for macaques to exhibit cultural tendencies. These characteristics permit comparisons with other technological, nut-cracking primates, such as chimpanzees and capuchin monkeys (Visalberghi *et al.* 2015). The comparison with chimpanzees goes further, however. As a result of the global expansion of oil palm monoculture, the same nut species, *Elaeis guineensis*, is exploited by stone tool using wild chimpanzees (*Pan troglodytes verus*) in Guinea (Biro *et al.* 2003; Humle and Matsuzawa 2004), alongside human populations that crack the same nuts (Sugiyama and Koman 1979). Investigating the similarities and differences in nut-cracking behavior, as well as the tools themselves, of primates using the same technology to access the same food source will allow the development of hypotheses regarding the evolution of hominin percussive technology. Percussive technology played an important role in the subsistence strategies of our early ancestors (Arroyo and de la Torre 2016; de la Torre and Mora 2005), and the earliest percussive behavior at Lomekwi 3 in West Turkana, Kenya, dated to 3.3 million years ago (Harmand *et al.* 2015), suggests that this behavior may span several hominin taxa. Identifying contemporary interspecies differences and similarities in the archaeological signature of primate percussive behavior may assist in developing hypotheses regarding the characteristics of hominin stone tool technology before the advent of the archaeological record.

## Data Availability

The datasets generated during the current study are not publicly available because of ongoing analysis using the same dataset but are available from the corresponding author on reasonable request.

## Electronic Supplementary Material

ESM 1 (MP4 29,596 kb)

ESM 2 (PDF 11519 kb)
