# Supplementary figures and images for: Technological Response of Wild Macaques (Macaca fascicularis) to Anthropogenic Change
Source: Int J Primatol. 2017 Aug 29;38(5):872–80. doi: 10.1007/s10764-017-9985-6 (PMC5629225; doi:10.1007/s10764-017-9985-6)

Anvil 2 - 3D Model

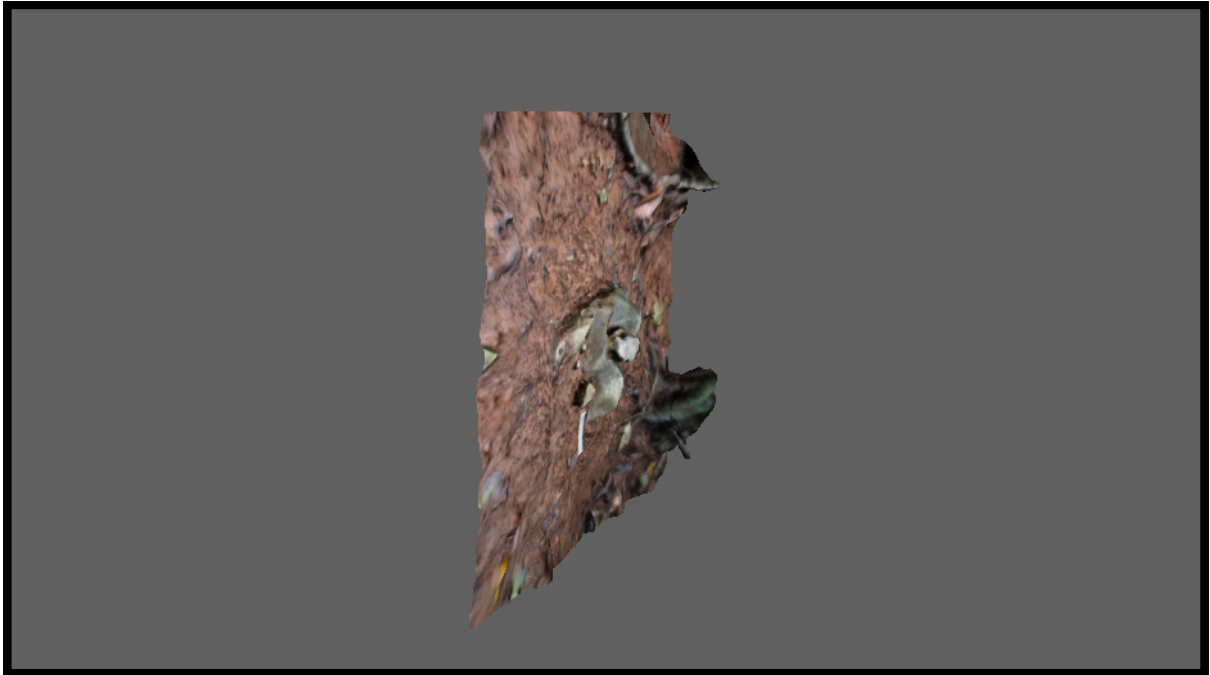

Anvil 2 - 3D Model Video

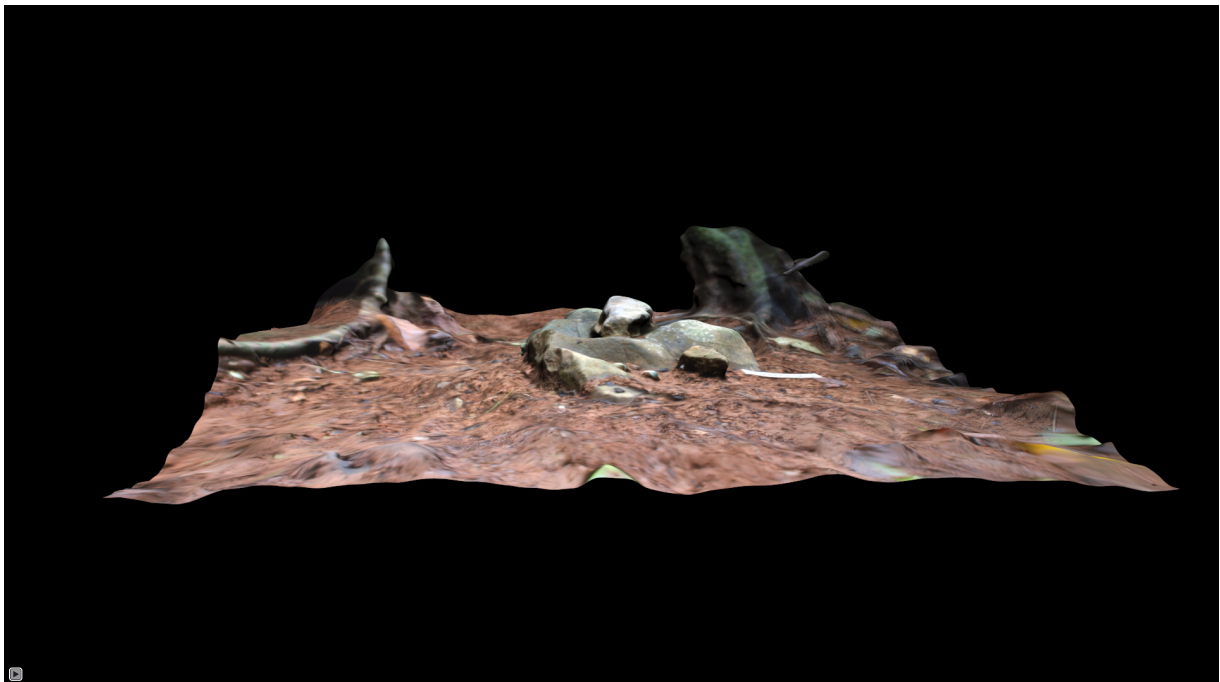

Supplement: Supplementary file 2 — (PDF 11519 kb) [file 10764_2017_9985_MOESM2_ESM.pdf]
